# An Overview of Glycosylation and its Impact on Cardiovascular Health and Disease

**DOI:** 10.3389/fmolb.2021.751637

**Published:** 2021-11-16

**Authors:** Karen Julissa Loaeza-Reyes, Edgar Zenteno, Adriana Moreno-Rodríguez, Rafael Torres-Rosas, Liliana Argueta-Figueroa, Roberta Salinas-Marín, Lizet Monserrat Castillo-Real, Socorro Pina-Canseco, Yobana Pérez Cervera

**Affiliations:** ^1^ Centro de Estudios en Ciencias de la Salud y la Enfermedad, Facultad de Odontología, Universidad Autónoma Benito Juárez de Oaxaca, Oaxaca, Mexico; ^2^ Centro de Investigación Facultad de Medicina-UNAM-UABJO, Universidad Autónoma Benito Juárez de Oaxaca, Oaxaca, Mexico; ^3^ Facultad de Medicina, Universidad Nacional Autónoma de México, Mexico City, Mexico; ^4^ Facultad de Ciencias Químicas, Universidad Autónoma Benito Juárez de Oaxaca, Oaxaca, Mexico; ^5^ Conacyt - Facultad de Odontología, Universidad Autónoma Benito Juárez de Oaxaca, Oaxaca, Mexico; ^6^ Laboratorio de Glicobiología Humana y Diagnóstico Molecular, Centro de Investigación en Dinámica Celular, Instituto de Investigación en Ciencias Básicas y Aplicadas, Universidad Autónoma del Estado de Morelos, Cuernavaca, Mexico

**Keywords:** glycosylation, O-GlcNAcylation, N-glycosylation, cardioprotective, hypertrophy, O-glycosylation, cardiovascular disease

## Abstract

The cardiovascular system is a complex and well-organized system in which glycosylation plays a vital role. The heart and vascular wall cells are constituted by an array of specific receptors; most of them are *N-* glycosylated and mucin-type *O-*glycosylated. There are also intracellular signaling pathways regulated by different post-translational modifications, including *O-*GlcNAcylation, which promote adequate responses to extracellular stimuli and signaling transduction. Herein, we provide an overview of *N*-glycosylation and *O-*glycosylation, including *O-*GlcNAcylation, and their role at different levels such as reception of signal, signal transduction, and exogenous molecules or agonists, which stimulate the heart and vascular wall cells with effects in different conditions, like the physiological status, ischemia/reperfusion, exercise, or during low-grade inflammation in diabetes and aging. Furthermore, mutations of glycosyltransferases and receptors are associated with development of cardiovascular diseases. The knowledge on glycosylation and its effects could be considered biochemical markers and might be useful as a therapeutic tool to control cardiovascular diseases.

## Introduction

Cardiovascular diseases (CVDs) are heart and circulatory system disorders, their most common reason is atherosclerosis (Sarre-Álvarez et al., 2018). One of the many risk factors involved in the development of CVDs is the metabolic syndrome: abdominal obesity, impaired glucose tolerance, hypertriglyceridemia, decreased high-density lipoprotein (HDL), high cholesterol, hypertension; the combination of any of these comorbidities increases the severity and the development of the atherosclerotic cardiovascular disease (Sperling et al., 2015; Stanley et al., 2009); other factors are lifestyle (tobacco smoking, sedentary lifestyle), age, and sex. At the cellular level, metabolic health is necessary to maintain cellular functions, including homeostasis, proliferation, and cellular activation. The alterations in metabolic signaling underlie a wide variety of diseases, including CVDs (Sousa Fialho et al., 2019). Another critical factor in the pathogenesis of CVDs is inflammation (Libby et al., 2011; Weber and Noels, 2011; Khambhati et al., 2018). Although it is still a poorly explored and complex area, some authors have described a link of metabolism with inflammation and atherosclerosis (Ali et al., 2018).

It is necessary to study the pathogenic mechanism to develop appropriate diagnoses and generate punctual therapies to resolve this complex disorder represented by CVDs (Yeh and Bickford, 2009). Fortunately, the development of technical advances in high-performance metabolomics profiling, nuclear magnetic resonance, and mass spectrometry has allowed for the identification of post-translational modifications; one of the most important is the protein glycosylation profile associated with different diseases (Akinkuolie et al., 2014; Otvos et al., 2015; Vercoutter-Edouart et al., 2015; Würtz et al., 2015; Connelly et al., 2016; Lauc, 2016; Lawler et al., 2016; Lawler and Mora, 2016; Connelly et al., 2017; Jang et al., 2020). Glycosylation could contribute to elucidate the mechanisms involved in the genesis and/or progression of CVDs (Gajjala et al., 2015).

Glycosylation is the most frequent post-translational modification in proteins because about half of the known proteins in eukaryote cells are glycosylated. Changes in protein glycosylation have been associated with physiological and pathological events involving migration, tumor invasion, cell growth, differentiation, host-pathogen interaction, cell trafficking, and transmembrane signaling (Ferrer et al., 2016).

There are two main types of protein glycosylation, the *N-* and the *O-*glycosylation. *O-*glycosylations are subdivided into mucin-type and *O*-GlcNAcylation. Growing evidence supports different roles for glycosylation in CVDs: 1) for example the *N-*glycans attached to different glycoproteins that exhibit a critical role for cardiovascular function show *N-*glycosylation, such as IgG glycans patterns, which allowed distinguishing patients with dyslipidemia from the controls (Liu et al., 2018); 2) changes in mucin-type *O-*glycosylation pattern in G protein-coupled receptors (GPCRs) are potential biomarkers in the pathogenesis of cardiac hypertrophy and heart failure (HF) (Nagai-Okatani and Minamino, 2016; Goth et al., 2020); and 3) *O*-GlcNAcylation that could be beneficial or impair cell functioning, dependent on the microenvironment (Ngoh et al., 2010; Jensen et al., 2019; Ng et al., 2021). This review aims to bring together research on the impact of *N*-glycosylation and *O*-glycosylation, including *O-*GlcNAcylation, on cardiovascular diseases.

## Glycosylation

Glycosylation is a co- and post-translational process where carbohydrates are covalently attached to carbohydrates, polypeptides, lipids, polynucleotides, usually catalyzed by glycosyltransferase enzymes, using specific substrates of sugar-nucleotide donors (Seeberger, 2017). Most glycan structures are found on the cell surface, such as *N-* and *O*-glycosylation, but can also be detected intracellularly like the *O*-GlcNAcylation. Glycosylation is a tightly controlled process involving glycosyltransferases and glycosidases that form the carbohydrate structures dependent upon sugar precursors, cellular environment, and cell type (Ohtsubo and Marth, 2006).

### *N*-Glycosylation

**FIGURE 1:**
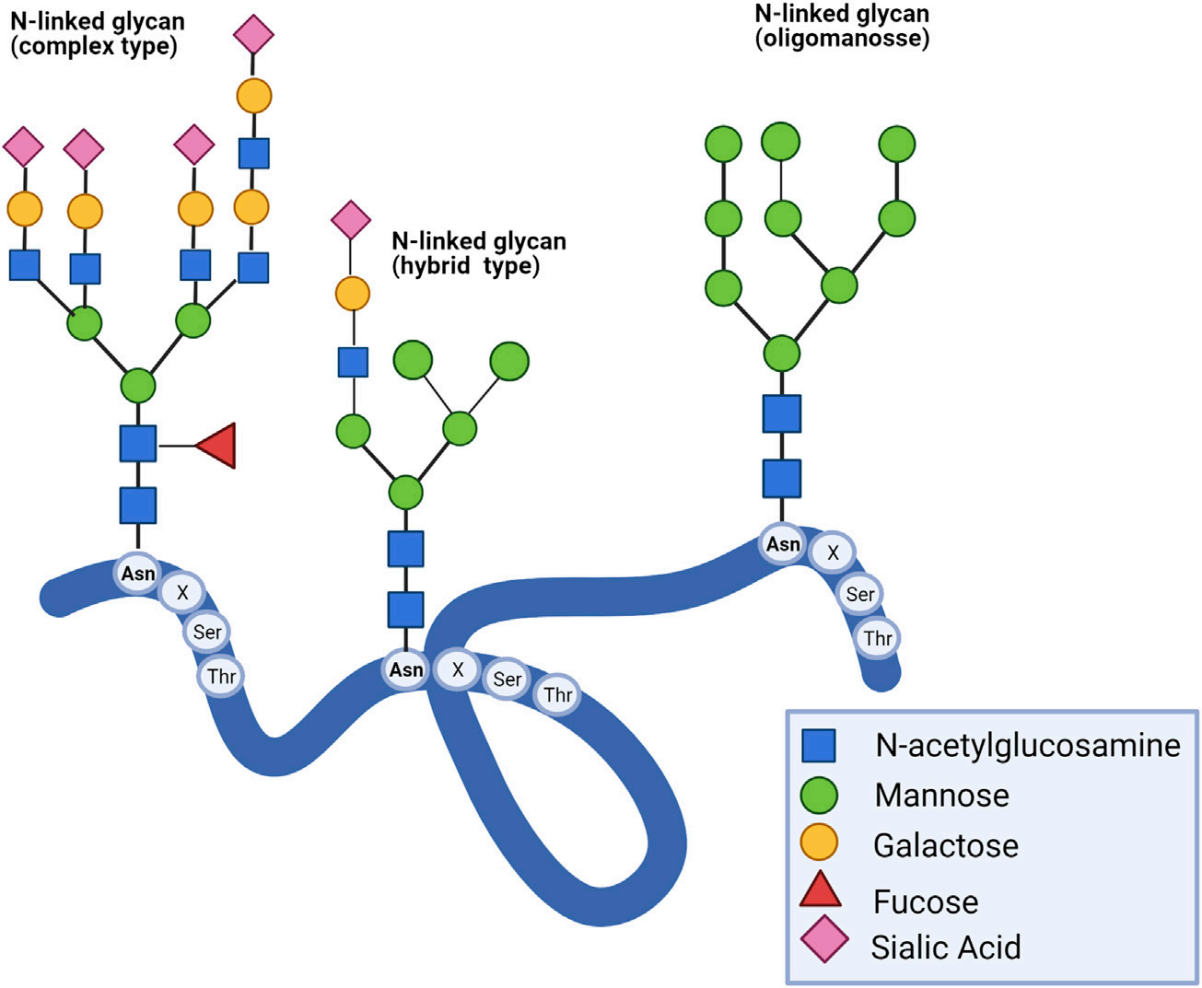
Type of N-glycans. There are three types of N-glycans: complex, hybrid, and oligomannose. All eukaryotic N-glycans share a consensus core sequence Manα1-6(Manα1-3) Manβ1-4GlcNacβ1-4GlcNac β1-Asn-X-Ser/Thr (Stanley et al., 2009).

*N-*glycans are covalently linked to polynucleotides and polypeptides on the asparagine residue by an *N*-glycosidic bond. The most common is *N*-acetylglucosamine bound to asparagine (GlcNAcβ1-Asn). The peptidic sequence to be glycosylated contains asparagine followed by another amino acid, except for proline, and ends with serine or threonine residues (Asn-X-Ser/Thr). A typical pentasaccharide core sequence shared in all *N*-glycans is: Manα1-6(Manα1-3)Manβ1-4GlcNacβ1-4GlcNac β1-Asn-X-Ser/Thr, and they have been categorized into three types: 1) oligomannose, in which only mannose residues are added to the core, 2) complexes in which antennas initiated by *N-*acetylglucosamine are attached to the core with sialic acid commonly found as terminal residue, and (Stanley et al., 2009) hybrid, in which only mannose residues are attached to the Manα1-6 arm of the core and one or two GlcNAc are added to the Manα1-3 arm (Figure 1) (Connelly et al., 2017). Complexity and diversity of *N*-glycan structures and their implications in disease progression are still a challenge.

### *O*-Glycosylation: Mucin-type

**FIGURE 2:**
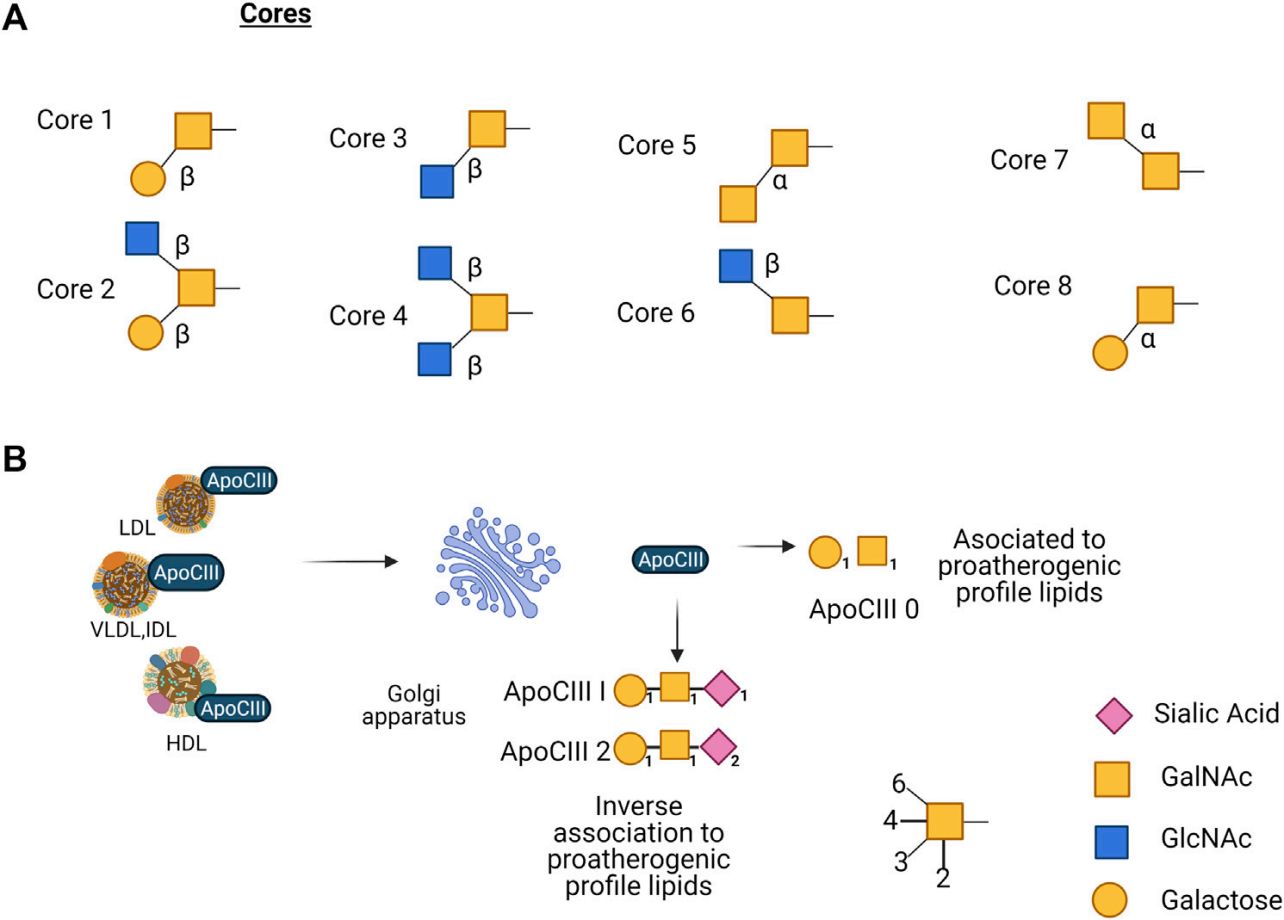
**(A)** Core structures reported of mucin-type *O*-glycans, eight in total, only cores 1–4 show widespread abundance, core structures 5–8 have a highly restricted occurrence, and core 7 has not been found in humans. **(B)** ApoC-III present in LDL, VLDL, and HDL is glycosylated in the Golgi apparatus, apoCIII_0_ (non-sialylated) is associated with proatherogenic profile lipids; apoCIII_1_ (mono-sialylated) and apoCIII_2_ (di-sialylated) are inversely associated with proatherogenic profile lipids.

*O-*glycosylation initiates by adding *N*-acetylgalactosamine (GalNAc) to the hydroxyl on some serine or threonine residues. It occurs in proteins that pass through the Golgi compartment, by an *N*-acetyl galactosaminyltransferase (GalNAc-T) that transfers the GalNAc residue to the side chain of a serine or a threonine. Until this date, 15 distinct members of the GalNAc-T family have been identified and characterized in mammals and as many as 24 isozymes that could be expressed in a tissue-specific manner (Kitada et al., 2013). The main structure of the core-α-GalNAc residue subsequently can be substituted at C3, C6, or at both positions with the monosaccharides β-galactose (Gal) at C3, β-GlcNAc at C3 and/or C6, and α-GalNAc at C3 or C6, producing 8 different cores by the action of glycosyltransferases. Although eight core *O*-glycan structures have been identified, only cores 1 to 4 show widespread abundance. The other core structures 5 to 8 have a highly restricted occurrence, and core 7 has not been found in humans (Brockhausen et al., 2017). Subsequently repetitive disaccharide elements, Galβ1-4GlcNAc, are added to the core, either in a linear or branched way. The backbones can suffer elongation forming complex structures by acetylation, sialylation, sulfatation, fucosylation, and polylactosamine extension (Hanisch, 2001; Steen et al., 1998) (Figure 2A). When *O*-glycosylation is added to proteins, it impacts different cell processes like transport of glycoproteins, protein conformation, resistance to proteolysis, and cell-cell and cell-matrix interaction (Breloy and Hanisch., 2018).

### *O*-GlcNAcylation

**FIGURE 3:**
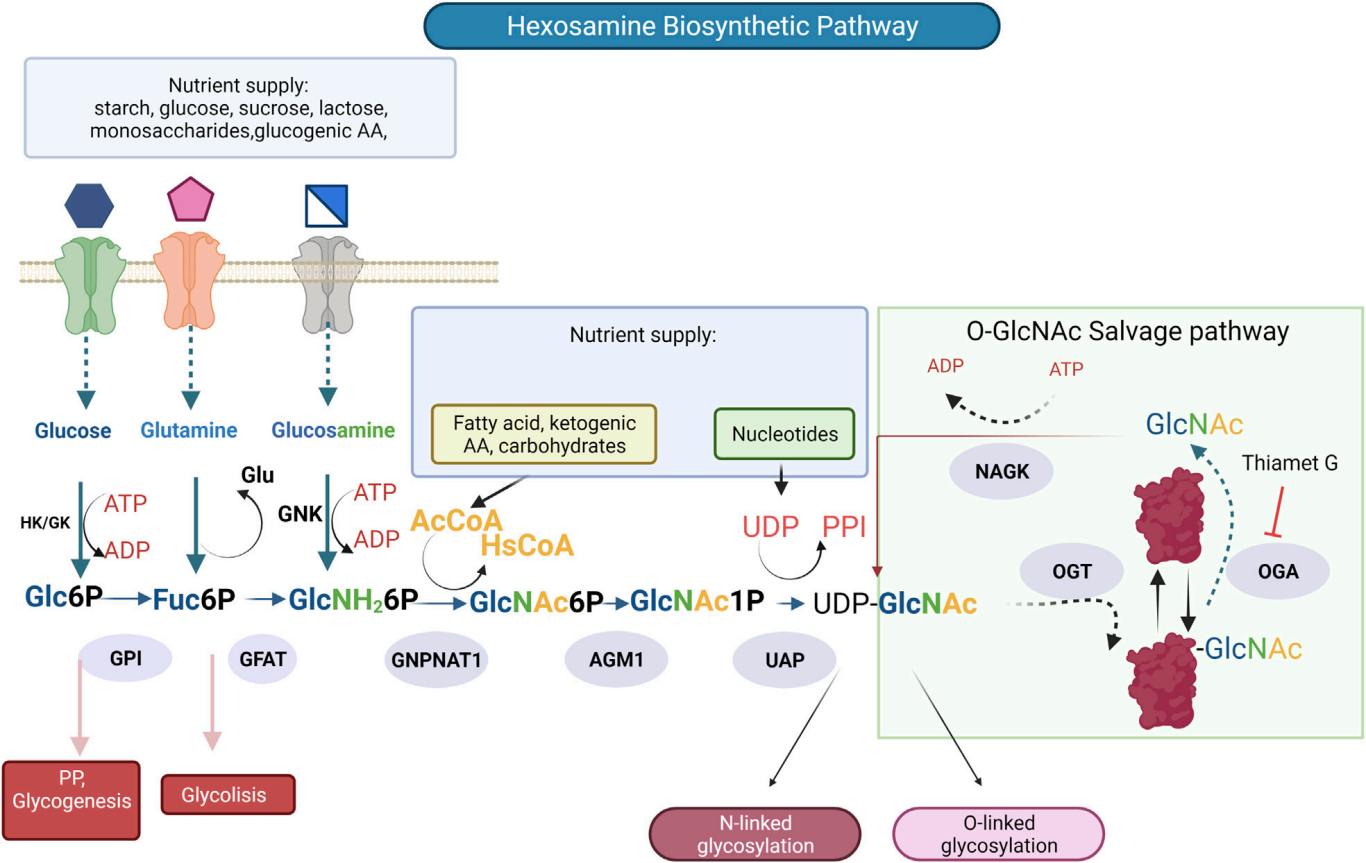
The hexosamine biosynthetic pathway (HBP) is responsible for the production of UDP-N-acetylglucosamine (UDP-GlcNAc), the substrate for protein glycosylation. Glucose is phosphorylated by hexokinase (HK) producing glucose-6-phosphate, which is converted into fructose-6-phosphate by phosphoglucose isomerase (GPI). At this point (F6P) can come into the HBP or continue through glycolysis to produce pyruvate. In the rate limiting step of the HBP (GFPT1/GFAT) -glutamine-fructose-aminotransferase catalyzes the conversion of fructose-6-phosphate and the amino group donor, glutamine to glucosamine-6-phosphate (GlcN-6P) and glutamate (Glu). The subsequent reaction is catalyzed by glucosamine-6-phosphate N-acetyltransferase (GNPNAT1) transform glucosamine-6-phosphate and acetyl-coenzyme A (Ac-CoA) to N-acetylglucosamine-1-phosphate (GlcNAc-1P) and CoA. Then, the phosphoglucomutase 3 (PGM3/AGM1) converts (GlcNAc-6P) to N-acetylglucosamine-1-phosphate (GlcNAc-1P). The following enzymatic reaction UDP-N-acetylglucosamine pyrophosphorylase 1(UAP1) utilizes GlcNAc-1P and UTP for the synthesis of uridine diphosphate *N-*acetylglucosamine (UDP-GlcNAc), Enzymes are involved (blue ovals), Thiamet G is an inhibitor of enzyme O-GlcNAcase.

Another type of *O-*glycosylation is the post-translational modification by *O*-GlcNAcylation (*O-*GlcNAc) was discovered in 1983 (Torres and Hart, 1984). *O*-GlcNAcylation is a dynamic post-translational modification generated by addition of β-D-N-acetylglucosamine onto serine (Ser) or threonine (Thr) residues, in nuclear, cytoplasmic, and mitochondrial elements (Baudoin and Issad., 2014); unlike other glycosylations, GlcNAc is not prolonged in complex structures. *O*-GlcNAcylation alters the functional properties of modified proteins, including transcription factors and epigenetic regulators. Because this modification depends on the glucose concentration, it constitutes a mechanism to regulate the activities of proteins according to the availability of glucose (Issad et al., 2010). It is reversible, highly dynamic, and interacts dynamically with phosphorylation. Two enzymes regulate *O*-GlcNAcylation: *O*-GlcNAc transferase (OGT) and *O-*GlcNAc hexosaminidase (OGA), whose donor substrate is UDP-*N*-acetylglucosamine (UDP-GlcNAc) (Olivier-Van Stichelen et al., 2012). *O-*GlcNAcylation can be considered a transverse regulatory mechanism, “rheostat”, that could control the intensity of the signal through different pathways according to the nutritional status of the cell: being the activity of OGT dependent on the concentration of UDP-GlcNAc (Issad et al., 2010), which is produced by the hexosamine biosynthetic pathway (HBP), this substrate is also used in the synthesis of *N-* and *O*-glycans mucin-type, (Figure 3) (Lam et al., 2021). Recently, Olson et al. found that glucose metabolism *via* the HBP was only ∼0.006% of the glycolytic efflux in *ex vivo* mouse heart (Olson et al., 2020), this is a value lower than the cited estimate of 2–3% of glucose uptake consumed by the HBP, calculated from cultured adipocytes (Marshall et al., 1991). Other metabolites like glucosamine (GlcN), glutamine, and acetyl coenzyme are converted also through the HBP to UDP-*N*-acetylglucosamine (UDP-GlcNAc). HBP is considered a unifying metabolic sensor that integrates carbohydrates, fatty acids, amino acids, nucleotides, and energy metabolism to produce UDP-GlcNAc and can be altered under physiological and pathological conditions (Ong et al., 2018).

## *N-*Glycosylation Regulates Crucial Functions in Cardiomyocytes

*N-*glycosylation modulates interactions of receptors and ligands with themselves, coregulatory molecules, and distinct membrane domains of intact cells, thereby altering signal transduction (Ohtsubo and Marth, 2006). The processing of many receptors is mediated by *N-*glycosylation (Klaver et al., 2019), receptors involved in cardiac functions are not the exception; corin and furin are cardiac transmembrane serine proteases that are *N*-glycosylated, which process their respective pro-natriuretic peptides (pro-NPs) into *O*-glycosylated active natriuretic peptides (Ichiki et al., 2013) involved in control of cardiac output, where *N*-glycosylation regulates their subcellular localization and biologic activity. The reduction in corin activity is due to a direct effect of *N*-glycosylation independently of its subcellular localization (Gladysheva et al., 2008); mutations in two binding sites for *N*-glycosylation in the protease domain of corin prevent its activation in HEK 293 and HL-1 cells (Liao et al., 2007; Gladysheva et al., 2008). Furin presents also *N*-glycosylation, although unlike corin, glycosylation is not essential for autocatalytic processing of profurin (Creemers et al., 1995). Another example where *N*-glycosylation is involved in cardiomyocyte function is the subunit α2δ1 of the Ca2+ channel, which is needed to maintain the basic cellular electrophysiological properties and control cardiac contractility. This function seems to be regulated by hybrid/complex *N*-glycosylation, and reduction of this process is sufficient to lead to dilated cardiomyopathy and early death in mice with depleted mannosyl (α-1,3-)-glycoprotein β-1,2-*N*-acetylglucosaminyl transferase (Ednie et al., 2019a; Ednie et al., 2019b).

Catecholamines are hormones that include epinephrine and norepinephrine (NE) that act in the heart through adrenergic receptors, beta 1 and beta 2. The β1-adrenoceptor (β1AR) is a GPCR that mediates cardiac contractility and the contraction force, prolonged receptor stimulation results in a reduction in beta-receptor sensitivity. These receptors, which transduce signals derived from catecholamine binding to a cellular response, have garnered considerable interest as therapeutic targets because of their key roles in the physiological control of cardiovascular functions, in the pathogenesis of cardiac arrhythmias, ventricular remodeling, and the evolution of HF (Park et al., 2017); high and rising catecholamine levels are associated with worsening of the prognosis in patients with heart failure. Catecholamines aggravate cardiac damage in ischemia, excessively high catecholamine concentrations cause myocardial damage in normal hearts (Prichard et al., 1991). The adrenergic nervous system and circulating catecholamines play an important role in the normal physiology and pathophysiology of the heart (Lefkowitz et al., 2000). NE is released in response to hypotension, and it is routinely administered in clinical settings to treat moderate to severe hypotension that may occur during general anesthesia and shock states (Chignalia et al., 2020). There is evidence that an increased cardiac release of NE and depleted cardiac stores of NE are two relevant features of the human failing heart, the decrease of NE uptake is caused by a reduction of the NE transporter density and this is induced by the actions of oxidative metabolites of exogenous NE, involving endoplasmic reticulum stress and impaired *N*-glycosylation of the NE transporter (Mao et al., 2005; Liang, 2007). With the use of tunicamycin, an *N*-glycosylation inhibitor, it has been shown that *N*-glycosylation is important for biosynthetic maturation, stability, and functional expression of human NE transporter (Melikian et al., 1994).

### *N*-Glycosylation is implicated in Cardiovascular Risk

**FIGURE 4:**
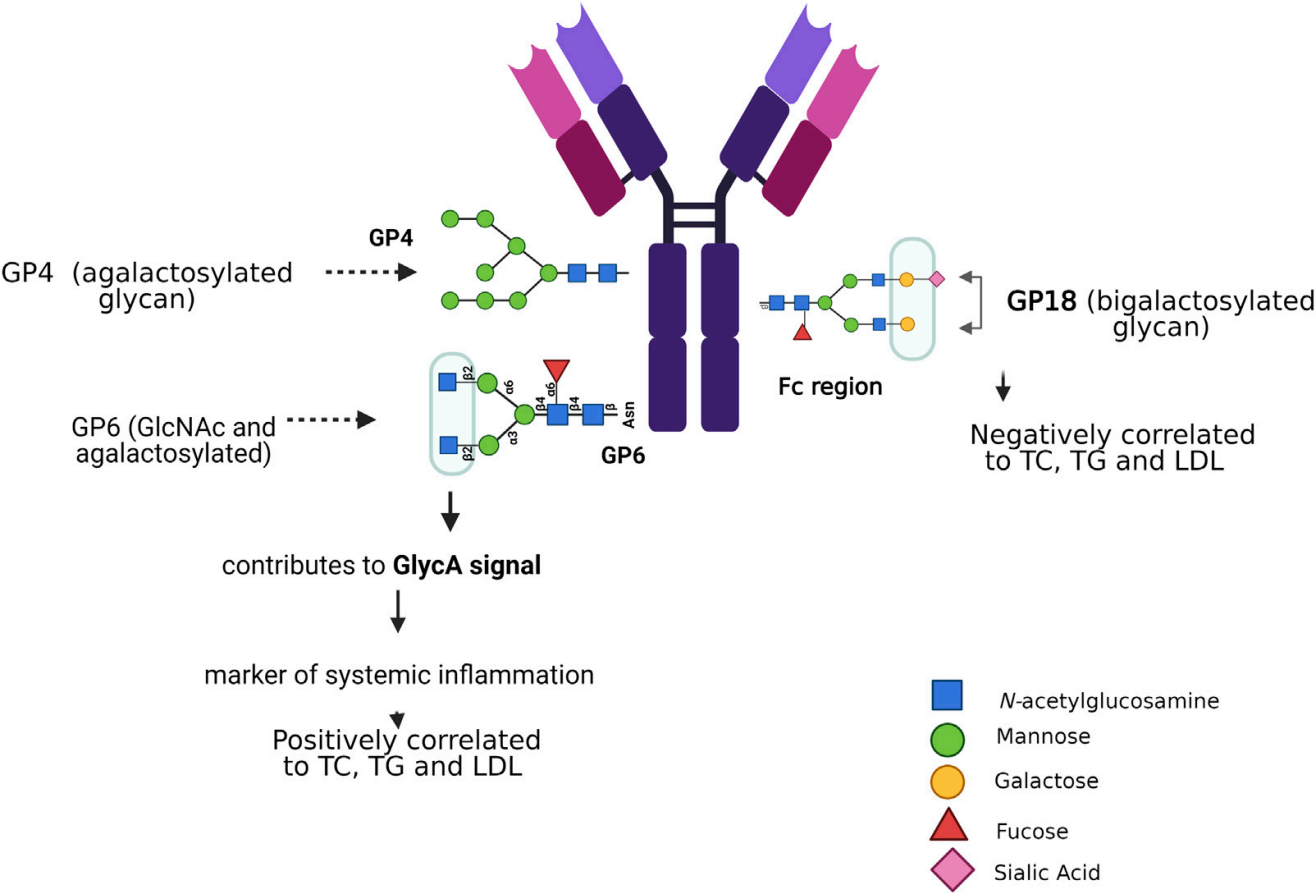
*N-*glycosylation associated with risk factors for dyslipidemia. *N-*glycans that attach to the Fc portion of IgG modulate the effector functions of IgG. Agalactosylated glycans and bisecting GlcNAc are positively correlated with elevated lipid levels, unlike sialylated and bigalactosylated glycans that are negatively correlated with TC, TG, and LDL. Characterizing the serum glycome could be a novel tool to identify markers and potential mediators of CVDs.

There are some risk factors related to *N*-glycosylation that contribute to cardiovascular risk, like sex, age, systolic blood pressure, total and HDL-cholesterol, and diabetes mellitus type 2; they are discussed below (Gudelj and Lauc, 2018). By evaluating the glycoproteome of the heart tissue at different ages in mice, it was demonstrated that high-mannose *N*-glycans increases with age, as well as an age-related regulation of GDP-mannose pyrophosphorylase, this enzyme facilitates the supply of the sugar-donor GDP-mannose (Franzka et al., 2021). The *N*-glycosylation in the Fc portion of IgG has been associated with age and the effect of estradiol, progesterone, and thyroid hormones, as shown in patients with thyroid cancer. These findings highlight the great impact of Fc glycosylation on IgG functions, which could contribute to the inflammatory immune response (Chen et al., 2012). Aberrant *N*-glycosylation has been related to gestational, type 1, and type 2 diabetes (Rudman et al., 2019), inactive *N*-acetylglucosaminyl transferase-IVa causes aberrant *N*-glycosylation and leads to impaired expression of GLUT2 glycosylation and glucose sensor function of beta cells, and, consequently, evokes type 2 diabetes in high fat diet mice (Ohtsubo et al., 2013; Ohtsubo, 2010). The insulin receptor (IR) beta subunit has been investigated through site-directed mutagenesis of each of the four asparagines, which participate in *N*-glycosylation sites; all mutant receptors are expressed normally at the cell surface, bind insulin with similar affinity, but have a beta subunit of smaller molecular mass; however, they showed defective induced internalization as compared to the wild type receptor (Leconte et al., 1994). *N*-glycosylation is correlated with insulin resistance, the latter was explored in obese mice, induced by a high fat diet. Hyposialylated IgG caused endothelial activation of the Fcγ receptor IIB, supplementation with the sialic acid precursor *N*-acetyl-d-mannosamine restored IgG sialylation and preserved insulin sensitivity without affecting weight gain (Reily et al., 2019; Tanigaki et al., 2017). Immunoglobulin G is a protein modified by *N*-glycosylation, its glycan profile has been associated with risk factors for dyslipidemia (Liu et al., 2018). *N*-glycans that are bound to the Fc portion of IgG are essential modulators of effector functions of IgG; thus, small changes in the glycosylation of IgG can lead to pro- and anti-inflammatory effects (Chan and Carter, 2010; Böhm et al., 2012). Glycosylation of IgG has been proposed as a biochemical marker in various inflammatory diseases, including hypertension (Gao et al., 2017). Analyses of plasma samples from 4,757 individuals of Chinese Han, Croatian, and Scottish ethnicity revealed lower expression of five digalactosylated IgG structures associated with prehypertension and hypertension; moreover, other 17 glycan traits were significantly related with hypertension (Wang et al., 2016). Liu et al. correlated the composition of 24 IgG *N*-glycan peaks (GPs) analyzed by HILIC–UPLC columns and blood lipid concentrations of total cholesterol (TC), total triglycerides (TG), HDL, and low-density lipoprotein (LDL). They found that TC, TG, and LDL were positively associated with the levels of GP4 and GP6, and negatively correlated with the level of GP18. Associations between blood lipids and the glycome of IgG were independently significant, with a negative association with loss of galactose and sialic acid, and a positive association with addition of a bisecting GlcNAc, IgG glycan patterns enabled distinguishing patients with dyslipidemia from controls (Figure 4) (Liu et al., 2018). In other studies, using nuclear magnetic resonance, the GlycA signal was observed in serum or blood plasma (Dierckx et al., 2019). The measured amplitude of the GlycA signal serves as a possible marker of systemic inflammation to detect the levels and states of glycosylation present in multiple inflammatory proteins; in fact, GlycA is associated with cardiovascular risk, diabetes mellitus, and mortality (Lawler and Mora, 2016).

The HDL receptor, Scavenger Receptor Class B Type 1 (SR-B1), mediates the transfer of HDL lipids to healthy cells; SR-BI overexpression has been found in cancerous tissues as it also mediates the transfer of cholesterol between HDL and malignant cells (Connelly and Williams, 2004; Mooberry et al., 2016). SR-BI has 11 potential *N-*linked glycosylation sites, mediates the selective cellular absorption of cholesteryl esters from HDL. In COS M6 cells transfected for wild-type or mutant SR-BI, the interruption of two sites, Asn-108 and Asn-173, alters the normal expression of the surface and the efficient absorption of lipids, however, it does not modify the binding affinity of the receptor to HDL (Babitt et al., 1997; Viñals et al., 2003). The glycosylation of HDL and LDL lipoproteins has functional implications in cholesterol metabolism; it has been concluded that the *N-*glycosylation of human plasma lipoproteins reveals a high level of diversity, which affects the functional properties of lipoproteins (Sukhorukov et al., 20191864). The native HDL shows significantly higher efficiency in removing cellular cholesterol from THP-1 cells compared to desialylated HDL by the neuraminidase treatment (*p* < 0.05) (Sukhorukov et al., 20191864; Pirillo et al., 2021).

Independently from the genetic or lifestyle habits origin of aberrant glycosylation associated with CVDs, characterizing *N-*glycoproteome in health and disease can lead to improved diagnoses and therapy. In this way, IgG *N-*glycosylation may participate in the pathogenesis of CVDs regulating the Fc portion of IgG promoting the pro- or anti-inflammatory response of IgG. Further analyses are necessary to advance in understanding the GlycA as a key marker of systemic inflammation and make this test available to clinical areas. Characterization of post-translational modifications involved in health and CVDs using technologies like mass spectrometry and selected reaction monitoring/multiple reaction monitoring help to develop early diagnosis and develop therapeutic drugs (Yang et al., 2015a; Gianazza and Banfi., 2018).

## Mucin-type *O*-Glycosylation and Congenital Disorders of Glycosylation

Although it is not the purpose of this review, we must mention that the congenital disorders of glycosylation (CDG) are a genetically and clinically heterogeneous group of over a hundred diseases, caused by defects in the synthesis and attachment of glycans to glycoproteins and glycolipids (Péanne et al., 2018). The originally known mutations were glycosyltransferases, remodeling glycosidases, and sugar nucleotide transporters, nevertheless recently new forms of CDG have been described with defects in vesicular trafficking, pH homeostasis or Mn^2+^ homeostasis. CDG are systemic syndromes that impact the brain, liver, eyes, muscles, skeleton, cardiac diseases, facial dysmorphisms, among others (Kościelak, 2005). CDG are classified into defects in *N*-glycosylation, *O*-glycosylation, glycosphingolipid and glycosylphosphatidylinositol anchor glycosylation defects, and multiple glycosylation pathway defects (Kościelak, 2005; Bogdańska et al., 2021).

### *O-*Glycosylation in Cardiomyocytes

*O*-glycosylation has a role in many biological processes, like pathogen interaction (Ricciuto et al., 2008), cell adhesion (Lasky et al., 1992), and proteolytic processes (Peng et al., 2011), aberrant glycosylation has been related to multiple diseases (Stuchlová Horynová et al., 2013; Duarte et al., 2016; Darula and Medzihradszky, 2018; Shajahan et al., 2020). In cardiomyocytes under normal conditions, the family of NPs consists of atrial natriuretic and brain natriuretic peptides (BNP) that are synthesized in response to atrial cardiomyocyte stretch, increased natriuresis, diuresis, and vascular permeability; these actions result in decreased cardiac output (Schellenberger et al., 2006). proBNP is convertase-dependently processed into BNP, the biologically active C- part with 32 amino acid residues and NT-proBNP with 76 amino acid residues. ProBNP is *O*-glycosylated in the central region of the protein sequence, this glycosylation prevents the cleavage to form biologically active BNP (Schellenberger et al., 2006). In physiological conditions, glycosylation of proBNP-108 site, controls the stability and processing of extracellular proBNP-108; increases of BNP and NT-proBNP have been associated with adverse outcomes in HF patients (Semenov et al., 2009; Tonne et al., 2011). Inhibition of *O*-glycosylation by digestion with PNGase F, O-glycosidase, and sialidase A leads to decreased pro-BNP stability (Jiang et al., 2010). Glycosylation of proBNP at threonine 71 prevents cleavage to form bioactive BNP1-32 and NT-proBNP; in obese patients with chronic heart failure, it was demonstrated that proBNP, not glycosylated at threonine 71, was decreased; owning to this lack, authors concluded that *O*-glycosylation is relevant to reduce plasma concentrations of BNP/NT-proBNP in obesity (Lewis et al., 2020).

Several GPCRs are cleaved in an agonist-dependent manner, including β1AR (Goth et al., 2020). KO mice were used as a model to demonstrate that GalNAc-T2 co-regulates the metalloproteinase-mediated limited proteolysis of β1AR; aberrant *O*-glycosylation seems to enhance proteolysis allowing for receptor signaling attenuation (Goth et al., 2017), β1ARs contain an O-glycan-regulated ADAM17-dependent *N*-terminal cleavage site at S41↓L42 (Park et al., 2017), the β1AR signaling pathway emerges as a key actor during the progression of heart failure. Prominent irregularities in β-AR signaling changes include a reduction of the β1AR levels of up to 50% (Madamanchi, 2007). β1AR *O*-glycosylation sites implicate *O*-glycosylation as a mechanism that prevents β1AR N-terminal cleavage and influences β1AR signaling responses in cardiomyocytes (Park et al., 2017).

Another example of how aberrant *O*-glycosylation influences heart disease is the delayed rectifier potassium IKs channel, which is an important regulator of the duration of the ventricular action potential expressed in cardiomyocytes, composed of a pore-forming α-subunit of a voltage-gated potassium channel subunit and modulatory β (KCNE1) subunit. The function of KCNE1 is to produce the kinetic properties of the native IKs channel in response to sympathetic stimulation to ensure adequate diastolic filling time in the face of accompanying accelerated heart rate (Osteen et al., 2010). The KCNE1 is glycosylated at Thr 7 and this glycosylation seems to be required for an efficient traffic to the plasma membrane, a mutation in this site is associated with cardiac arrhythmias (Chandrasekhar et al., 2011). When physiological conditions are altered as is the case of salt-induced hypertension, the glycosyltransferases GALNT1, GALNT2, and GALNT7 enzymes are up regulated, leading to the aberrant *O-*glycosylation of the cysteine and glycine-rich protein 3 (Nagai-Okatani and Minamino, 2016). Studies in Galnt1-deficient mice showed cardiac dilation and valve thickening, evidencing that glycosyltransferase 1 is critical in the normal heart valve development and cardiac function (Tian et al., 2015).

### Mucin-type *O*-Glycosylation in HDL and LDL Lipoproteins

There is consistent evidence that high LDL-C concentrations increase the atherosclerotic cardiovascular risk as shown by randomized genetic studios and intervention trials (Ference et al., 2017), low HDL-C contributes to elevated cardiovascular risk (Chapman et al., 2011). The HDL exerts a critical function in transporting cholesterol from macrophages in peripheral tissues to the liver for elimination, it also carries out various functions such as antioxidants, stimulation of nitric oxide production in endothelial cells by activating surface receptors. The main protein of the HDL molecule is apolipoprotein A1, which is synthesized in the liver and intestine, apolipoprotein C-III (apoC-III) is present especially in very low-density lipoprotein and is also present in chylomicrons and HDL. High plasma concentrations of apoC-III has been associated with high concentrations of TG (Bayly et al., 2014; Ertek, 2017). Genes of glycosyltransferases, like the *GALNT2*, have been associated with modifications of plasma lipid concentrations and, consequently, with susceptibility to cardiovascular disease (Tran and Ten Hagen, 2013; Shukla et al., 2019). As a result of genetic variants of single nucleotide polymorphism (SNPs), an important association was reported between the glycosyltransferases, GALNT2, and the concentration of HDL cholesterol and TG, whereas B3GALT4 and B4GALT3 were associated with LDL-C, these glycosyltransferases could possibly modify a lipoprotein or receptor (WillerCristen et al., 2008). *GALNT2,* which codes for the *O*-glycosylation enzyme, ppGalNAc-T2, has been considered as a regulator of HDL-C and TG serum levels (Di Paola et al., 2017)**.** Furthermore, Galnt2-KO mice showed a reduction of HDL-C and high concentrations of TG, compared to the wild type, probably by regulating three specific targets, the angiopoietin-related protein 3 (ANGPTL3), ApoC-III, and phospholipid transfer protein (Zilmer et al., 2020), ANGPTL3 is considered a promising therapeutic target in patients with dyslipidemia who are at risk of atherosclerotic cardiovascular disease (Lim, 2017). An SNP of GALNT2 was also associated to hypertension in a Chinese population (Zhang et al., 2015a). Furthermore, the association of some SNPs of the GALNT2 with serum TG levels has been demonstrated in the Han ethnic group (Guo et al., 2016).

Another key regulator glycosylated apolipoprotein in TG metabolism is apoC-III, the mature apoC-III polypeptide undergoes glycosylation with Gal, GalNAc, and different molecules of sialic acid in the Golgi apparatus prior to its incorporation into lipoproteins. ApoC-III shows three glycoforms: non-sialylated (apoC-III_0_), monosialylated (apoC-III_1_), and disialylated (apoC-III_2_). Studies show an inverse association between apoC-III_2_ and proatherogenic plasma lipid profiles in individuals with abnormal glucose metabolism; this suggests that the relative amount of apoC-III glycoforms could be part of the risk assessment in patients with type 2 diabetes mellitus (DM2) (Koska et al., 2016; Yassine et al., 2015). Also, another study that investigated the correlation between glycoforms in plasma, apoC-III non-sialylated (apoC-III0a without glycosylation and apoC-III0b with glycosylation), monosialylated (apoC-III1) or disialylated (apoC-III2) proteoforms, revealed that apoC-III0a, apoC-III0b, and apoC-III1, but not apoC-III2 appear to be under metabolic control and associate with fasting plasma TG. Measurement of apoC-III proteoforms can offer insights into the biology of TG metabolism in obesity (Figures 2B) (Yassine et al., 2015).

The isoelectric focusing technique, applied to evaluate *O*- glycosylations in apoC-III, has been described for the complementary diagnosis of CDG (Wopereis et al., 2007) and, recently, mass spectrometry has been used to evaluate the glycosylation profile (Wada et al., 2012; Nicolardi et al., 2013a). Liquid chromatography coupled with time-of-flight and ion trap mass spectrometry has been utilized for the identification of a fucosylated apolipoprotein-CIII isoform (Nicolardi et al., 2013b)**.** As an example of diagnosis of GALNT2-CDG polymorphisms: GALNT2 loss-of-function is associated with decreased HDL-C levels in rodent KO- models (Zilmer et al., 2020).

*O*-glycosylation in cardiomyocyte proteins is involved in the correct functioning of the protein like stability and membrane trafficking, nevertheless aberrant glycosylations that originate from mutations in one of the glycosyltransferases, or that may be related to a mutation in the protein binding site, make this glycosylation failure a problem that is related to CVDs. In this review, some examples are proposed that can lead to a better understanding of the development of CVDs and, with more studies, glycosylations can become therapeutic targets or be used for early diagnosis.

## *O*-Glycosylation: Type *O*-GlcNAcylation on Heart and Vascular Wall

The first studies that reported the involvement of *O*-GlcNAc in the heart were done by Hart’s group in 1996, in which, using two-dimensional electrophoresis of rat heart samples, they found two *O*-GlcNAcylated forms of αβ-crystallin (Roquemore et al., 1996). Later, in 1997, Järvinen et al. (Yki-Järvinen et al., 1997) reported a higher activity of OGT in the rat heart, compared to other tissues (liver, fat, striated muscle). Currently, a wide variety of studies show the participation of *O*-GlcNAc in the heart and vasculature (Collins and Chatham, 2020). For example, *O*-GlcNAcylation shows dynamic and extensive cross-talk protein phosphorylation, including the mitogen-activated protein kinase (MAPK) pathway, and regulates various cellular signals and functions (Zhang et al., 2015b). Although some studies emphasized that *O-*GlcNAcylation provide significant cardio protection, higher *O-*GlcNAc levels increase vascular reactivity to constrictive stimulation, impair nitric oxide-dependent arteriolar dilations, reduce neointimal formation, prevent inflammation-induced vascular dysfunction, and modulate placental vasculogenesis (Xing et al., 2008; Yang et al., 2015b; Collins and Chatham, 2020). On the other hand, increased levels of *O-*GlcNAcylation may increase contractile responses in the rat basilar artery through activation of the MAPK pathway and, thus, may contribute to abnormal vascular reactivity; this was analyzed using Wistar rats, a group on a control diet (10% fat) and the other on a high-fat diet (45% fat) for 12 weeks (Lima et al., 2016).

Another factor that influences heart disease is inflammation, OGT mediates LPS-induced nuclear transcription factor kappa-beta-p65 (NF-κβ/p685) entry and activation of endothelial cells; this was proven using ST045849 (10 μM), a potent OGT inhibitor (Li et al., 2014). LPS treatment elevated mRNA levels of interleukin 1 beta, monocyte chemoattractant protein-1, and E-selectin in the vehicle (PBS), but not in OGT inhibitor-treated cells (Li et al., 2014). Additionally, preincubation with mTempol (a mitochondria-targeted antioxidant with superoxide scavenging properties) significantly reduced LPS-mediated NF-κβ nucleus translocation, supporting a potential cause-and-effect relationship between NF-κβ modification and nuclear entry, modulated by the superoxide via MAPK (Li et al., 2014).

**FIGURE 5:**
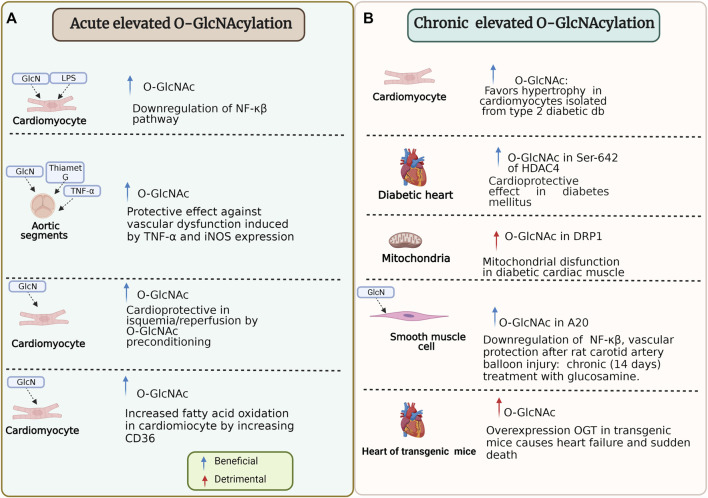
Beneficial and detrimental effects of O-GlcNAcylation due to the timing of the modification and specific stimulus. **(A)** Some experiments that reflect effects under specific conditions of acute elevated O-GlcNAcylation on cardiomyocyte and aortic segments. **(B)** Some experiments that reflect effects under specific conditions of chronic elevated O-GlcNAcylation on cardiomyocyte, mitochondria, smooth muscle cell, and heart function. HDAC4, histone deacetylase 4; NF-κβ/p65, nuclear transcription factor kappa-beta-p65; LPS, lipopolysaccharide; GlcN, Glucosamine; OGT, O-linked N-Acetylglucosamine transferase; TNF-α, tumor necrosis factor-α; iNOS, inducible nitric oxide synthase; O-GlcNAc, O-linked β-N-acetylglucosamine; DRP1, dynamin-related protein 1; A20, also known as TNFAIP3 tumor necrosis factor α-induced protein 3. For more detail see the text.

*O*-GlcNAc is capable of adapting itself to stress response because, in some conditions, it could be beneficial (e.g., ischemia/reperfusion) or, on the contrary, deleterious (e.g., diabetes) (Collins and Chatham, 2020; Chen et al., 2019; Bolanle et al., 2021; Wright et al., 2017). Then, *O*-GlcNAcylation has an ambivalent role in proteins (Figure 5). In this regard, *O-*GlcNAcylation of histone deacetylase 4 at Ser-642 is considered cardioprotective in type 1 and 2 diabetes mice model and counteracts pathological Ca^2+^/calmodulin-dependent protein kinase II signaling (Kronlage et al., 2019). However, other studies show that *O*-GlcNAc promotes hypertrophy in cardiomyocytes isolated from type 2 diabetic db/db mice and non-diabetic controls treated with 1 μM angiotensin II and 10 μM phenylephrine for 24 h (Marsh et al., 2011).

### The Role of *O*-GlcNAc and HBP in Contributing to the Adverse Effects of Diabetes on the Heart and Vasculature

Although *O*-GlcNAcylation has been known for more than 30 years, it has recently become important in most biological processes. This modification is found in most living organisms (fungi, bacteria, plants, animals) and, recently, described in protists (Aquino-Gil et al., 2018; Perez-Cervera et al., 2011), which may constitute an ancestral mechanism for regulating protein activity concerning the nutritional status of the cell. As previously described, there is an analogy between *O*-GlcNAcylation and phosphorylation (Van der Laarse et al., 2018). The levels of phosphorylation and *O*-GlcNAcylation are susceptible to the nutritional state of the cell, each with its respective donor, ATP and UDP-GlcNAc, high potential substrates, products of cellular metabolism. All the *O*-GlcNAcylated proteins identified to date can also be modified by phosphorylation, which reinforces the possible interaction between these two modifications (Van der Laarse et al., 2018; Mercier et al., 2018; Leney et al., 2017). On the other hand, *O*-GlcNAcylation regulates insulin signaling and plays an essential role in developing diabetes and its complications, like CVDs. Insulin resistance, defined as a decrease in the efficiency of insulin action in target tissues, is a characteristic of DM2. It is widely known that chronic hyperglycemia per se has deleterious effects on the sensitivity of insulin (Garvey et al., 1985). In 1991, Marshall et al. (1991) proposed a communication pathway between HBP and insulin resistance. They observed a marked decrease in insulin sensitivity in primary adipocytes that could be induced by a concomitant addition of glutamine, high glucose concentration, and insulin in the culture medium. The effect of these agents was inhibited by drugs that act on glutamine fructose-6-amidotransferase (GFAT), the rate-limiting enzyme of HBP. However, GlcN alone, which enters the pathway after GFAT (Figure 3), was much more efficient in inducing insulin resistance compared to adding all three agents together (Marshall et al., 1991). Based on the results previously described, the authors proposed that HBP played an essential role in insulin resistance (Marshall et al., 1991). The role of HBP and protein *O*-GlcNAcylation in insulin resistance was clearly demonstrated by McClain (2002), who developed transgenic mouse models, which overexpress GFAT or OGT in different tissues. The authors demonstrated that overactivation of the pathway in muscle, adipose tissue, liver, or pancreatic β cells resulted in phenotypes like those seen in DM2 and obesity. Specifically, the overexpression of GFAT in tissues involved in glucose uptake through insulin stimulation (adipose tissue and muscle) and that use the GLUT4 promoter produced insulin resistance associated with a decrease in the translocation of the glucose transporter induced by insulin. This insulin resistance can be reversed with the use of the antidiabetic drug, troglitazone (McClain, 2002). Another evidence of the involvement of *O*-GlcNAcylation in insulin resistance was obtained in transgenic mouse models when GFAT and OGT were overexpressed in muscle and adipose tissue, which use the same glucose promoter GLUT4, and observing disturbances in energy metabolisms, such as insulin resistance and hyperleptinemia, characteristics also observed in diabetic patients (Love and Hanover, 2005). This evidence allowed to carry out a more in-depth investigation on the *O*-GlcNAcylation of proteins involved in insulin signaling. More specifically, it is known that the binding of insulin to its receptor stimulates its autophosphorylation at tyrosine residues (Goodyear et al., 1995). This stimulates the receptor tyrosine kinase activity towards intracellular substrates, such as insulin receptor substrate 1 (IRS1) or Shc, which activate the signaling pathways involved in the mitogenic and metabolic effects of insulin (Combettes-Souverain and Issad, 1998). Immediately, many proteins involved in insulin signaling, from the plasma membrane to the nucleus, are *O*-GlcNAcylated, including the insulin receptor subunit, IRS1, IRS2, the subunits p85 and p110 of PI-3 kinase, PDK1, the protein kinase AKT/PKB, and the transcription factor FoxO1. In most cases, the *O*-GlcNAcylation of these proteins has effects opposite to those induced by insulin (Lefebvre et al., 2010). It has been shown that after insulin stimulation, OGT is directed towards the plasma membrane (Yang et al., 2008), specifically in the lipid microdomains (Perez-Cervera et al., 2013), around the first proteins involved in insulin signaling. In the basal state, OGT has an essentially cytosolic and nuclear location, and after stimulation by insulin, the production of phosphatidyl-inositol-triphosphate induces the recruitment of OGT in the plasma membrane through a domain called phosphatidyl-inositol-triphosphate binding domain of OGT, located in the C-terminal part of the enzyme. Recruitment of OGT to the membrane favors *O*-GlcNAcylation of insulin signaling pathway proteins resulting in attenuation of the signal. When cells are in an environment abnormally rich in glucose (and insulin), due to chronic hyperglycemia, as is the case of diabetic patients, this situation could be increased, which can lead to a decrease in the efficiency of insulin signaling and, therefore, to insulin resistance at the cellular level and the development of a vicious cycle (glucotoxicity) (Issad et al., 2010; Perez-Cervera et al., 2013), These first observations with respect to the insulin pathway allowed observing the adverse effects of glucose toxicity (Banerjee et al., 2016). Although the role of *O*-GlcNAc in the insulin signaling pathway in the heart is not known clearly, there are many *O*-GlcNAcylated proteins involved in diabetic complications associated with the heart; for example, in endothelial cells, the transcription factor, specificity factor 1 is *O*-GlcNAcylated in response to hyperglycemia linked to higher mitochondrial superoxide (Du et al., 2000), endothelial isoform of nitric oxide synthase (eNOS) is *O*-GlcNAcylated at ser 1,177, a primary phosphorylation-dependent activation site, resulting in endothelial cell dysfunction (Du et al., 2001; Wheatcroft et al., 2003; Kim et al., 2006). In cardiac muscle cells, dynamin-related protein 1 is *O*-GlcNAcylated at threonine 585 and 586, resulting in mitochondrial dysfunction (Gawlowski et al., 2012). There are many other studies in which diabetes increases *O*-GlcNAcylation, contributing to cardiac complications of diabetes (Clark et al., 2003; McNulty, 2007; Marsh et al., 2011; Fricovsky et al., 2012; Erickson et al., 2013; Marsh et al., 2013).

Other stimuli increase *O*-GlcNAcylation including insulin, leptin, endothelin 1, and phenylephrine, and others conditions like aging (Banerjee et al., 2016; Hart, 2019), in contrast, to acute and chronic exercise, in which *O*-GlcNAcylation decreases (Bennett et al., 2013; Cox and Marsh, 2013), although, the mechanism and effects on the heart are not clear.

CD36 or scavenger receptor B2, is a facilitator of energy in the heart, i.e., approximately 50–70% of lipid uptake (Kim and Dyck, 2016), is involved in CVDs: ischemia/reperfusion, diabetic cardiomyopathy, pathological cardiac hypertrophy, physiological cardiac hypertrophy, and atherosclerosis (Shu et al., 2020). CD36 is modified by a variety of posttranslational modifications (Luiken et al., 2016) including glycosylation. Ten potential *N*-linked glycosylation sites are within the CD36 protein sequence, and glycosylation explains the difference in molecular weight between the isolated protein (88 kDa) and the deduced cDNA sequence (53 kDa) (Abumrad et al., 1993). *N*-glycosylation in CD36 is necessary for the correct folding and membrane trafficking; however, glycosylation-site mutants do not alter ligand-binding (Hoosdally et al., 2009). Using a spontaneously hypertensive rats model, which simulates hypertension-induced left ventricular hypertrophy and metabolic syndrome, revealed that rats depict multiple variations in their CD36 sequence, this allowed identifying important aspects for the function of CD36 and its impact on the consumption of long-chain fatty acids in the heart, like that *O*-GlcNAcylation in the recruitment of CD36 to the membrane and *N*-glycosylation at asparagine 102 are crucial for the transport of long-chain fatty acids (Lauzier et al., 2011). Furthermore, acute regulation of cardiac metabolism by the stimulation of isolated rat hearts perfused with 0.05 mM GlcN leads to a significant increase of *O*-GlcNAc levels; GlcN treatment also increased markedly fatty acid translocase (FAT/CD36) levels in the membrane fraction in a concentration-dependent manner, this suggests that *O*-GlcNAcylation of CD36 induces CD36 translocation to the plasmatic membrane and fatty acid oxidation (Laczy et al., 2011). These results coincide with increases of FAT/CD36, higher LCFA uptake, and impaired insulin signaling, which reduced GLUT4 translocation, as observed in DM2 (Coort et al., 2007). The therapeutic potential of CD36 has been suggested although more studies are needed to fully understand signal transduction (Karunakaran et al., 2021). In this sense, the HBP and *O*-GlcNAcylation possibly represent a novel regulator of cardiac metabolism because acute modulation by the HBP and *O*-GlcNAc of heart proteins stimulate fatty acid oxidation, probably by increasing FAT/CD36 in the plasma membrane (Laczy et al., 2011). Also, OGT co-immunoprecipitated with CD36; this could be because CD36 is a target of *O*-GlcNAc. Another study suggests that increased fatty acid oxidation in the chronically stressed heart may be beneficial. NADPH oxidase 4 (Nox4) redirects glucose metabolism away from oxidation but increases fatty acid oxidation, maintaining cardiac energetics during acute or chronic stress. The glucose and fatty acid metabolism changes are interlinked via a Nox4-activating transcription factor 4 (Nox4-ATF4)-dependent increase in the HBP, which mediates the attachment *O*-GlcNAc to CD36 and enhances fatty acid utilization. These findings suggest that the Nox-4-ATF4-HBP signaling pathway is involved in adaptive metabolic reprogramming, which may contribute to the heart resisting pathological remodeling (Nabeebaccus et al., 2017).

Recently, Junfeng Ma et al. (Ma et al., 2016) reported 86 *O*-GlcNAcylated mitochondrial proteins, using comparative proteomics between control and streptozotocin induced diabetic rat hearts. It is important to know that mitochondria regulate cellular metabolism, which suggests that *O*-GlcNAcylation is a regulator of mitochondrial metabolism and could be a critical factor in the initiation and progression of diabetic cardiomyopathy. Among other proteins found is HSP60 that is *O*-GlcNAcylated. It is known that mitochondrial HSP60 facilitates the folding of mitochondrial proteins and prevents mitochondrial protein degradation (Duan et al., 2020). This protein also is involved in cardiovascular physiology and diseases (Duan et al., 2020).

### Cardioprotective Role of Acute Activation of *O*-GlcNAcylation in Heart and Vasculature

Cardiomyocyte death is the main cause of the impact on survival and quality of life (Severino et al., 2020) of ischemic heart disease (reduced blood flow in the heart), which occurs massively during acute myocardial infarction (Aydin et al., 2019). The development of treatments capable of restoring blood flow (reperfusion therapy) in patients with acute myocardial infarction has been the principal advance in this field. However, reperfusion therapy does not guarantee that ischemic cells survive, and, on the contrary, a part of cell death is precipitated by the restoration of the flow itself (Acharya, 2020). Therefore, it is necessary to search for and develop cardioprotective treatments that can improve the efficiency of reperfusion (Ruiz-Meana and García-Dorado, 2009). In this regard, the group of Champattanachai et al., in 2007, reported for the first time that the supply of 5 mM GlcN in rat neonatal cardiomyocytes improved cell viability after ischemia-reperfusion (I/R) injury and was correlated with an increase in *O*-GlcNAcylated proteins (Champattanachai et al., 2007). Later, Laczi et al., in 2010, reported that increasing the levels of *O*-GlcNAcylation, through the inhibition of OGA preserved cell viability during I/R (Laczy et al., 2010). At the same time, another group reported a novel regulator of cardiovascular function (Ngoh and Jones, 2008), the cardiac OGT expression, which is essential for the surviving myocardium in the failing heart, concluding that increments of *O*-GlcNAc attenuate ER stress-induced cardiomyocyte death in I/R (Watson et al., 2010; Ngoh et al., 2011); this cardioprotection mediated by *O*-GlcNAc is explained by the improved tolerance of mitochondrial oxidative damage and improved cell viability of cardiomyocytes (Jones et al., 2008; Darley-Usmar et al., 2012). Another study reported that upregulated protein *O*-GlcNAcylation and subsequent increases in the expression and translocation of members of the Bcl-2 protein family ameliorated the mitochondrial dysfunction and apoptotic cell death in I/R (Champattanachai et al., 2008).

Ischemic preconditioning (IPC) can be necessary to activate cardioprotection (Yellon et al., 1998) The *O*-GlcNAcylation shows potential association with cardioprotection by IPC (Jones et al., 2008; Jensen et al., 2013). In this sense, Pælestik et al. report the effects of hypoglycemia in IPC in hearts from rats with and without DM2, and they suggest that myocardial glucose uptake and *O*-GlcNAc levels are involved in the mechanisms of IPC (Pælestik et al., 2017).

Another aspect less explored is the role of *O*-GlcNAcylation on innate immunity in I/R injury and myocardial infarction. Innate immunity is beneficial in the heart in the short term by upregulating cytoprotective mechanisms, most notably mitochondrial stabilization, and facilitating tissue repair (Jensen et al., 2019). However, sustained toll-like receptors (TLR) signaling is maladaptive, leading to activation of circulating neutrophils and monocytes in peripheral circulation, which causes increased tissue destruction. Although the mechanisms that activate TLR signaling following tissue injury in the heart are not understood clearly, they involve signaling by proinflammatory cytokines and molecular patterns associated with damage (Spirig et al., 2012; Balistreri et al., 2017). For example, extracellular HSP60 selectively binds to the cardiac myocyte inducing cell death by apoptosis, when anti-TLR4 blocking antibodies decrease apoptosis suggests that HSP60 released during cardiac injury could have a paracrine effect on neighboring myocytes leading to cell death (Kim et al., 2009; Tian et al., 2013). It is known that HSP60 is *O*-GlcNAcylated in mitochondria (Ma et al., 2016); nevertheless, the role of *O*-GlcNAcylation in the function and localization of HSP60 in myocytes is still unknown. *O*-GlcNAcylated proteins could be emergent antagonists/inhibitors of the TLR signaling pathways to treat cardiovascular diseases (El-Zayat et al., 2019).

Recently, Priya Umapathi et al., using two models of transgenic mice in which OGT and OGA were overexpressed, found a severe development of cardiomyopathy and premature death in the transgenic heart with OGT, whereas in the transgenic heart with OGA, they found a decrease in *O*-GlcNAcylated proteins and an identical cardiac function to the control model. OGA transgenic hearts were resistant to pathological stress due to pressure overload, with a decrease in *O*-GlcNAcylation levels and a decrease in hypertrophy compared to wild-type controls (Umapathi et al., 2021). These results agree with those of Sujith Dassanayaka et al., who found that the OGA-deficient mice exhibited exacerbated cardiac function at 1 week following infarction (Dassanayaka et al., 2020). These findings suggest that *O*-GlcNAcylation represents a novel therapeutic approach for cardiomyopathy (Umapathi et al., 2021).

### Role of the *O-*GlcNAcylation in Cardiovascular Disease and Diabetes Mellitus: Focus on Inflammation

Cardiovascular disease and diabetes mellitus are linked. CVDs represent the principal cause of mortality and morbidity in diabetic patients (Nelson et al., 2019). Obesity, hypertension, and dyslipidemia are cardiovascular risk factors associated specifically with type 2 diabetes mellitus (DM2) (Rodgers et al., 2019). The vascular complications of diabetes are classified as macrovascular (lesions of larger blood vessels) and microvascular (lesions of small blood vessels). The macrovascular complications include CVDs, such as heart attacks, strokes, and circulatory failure in the lower limbs. On the other hand, the microvascular complications related to DM2 are retinopathy, nephropathy, and neuropathy (Beckman and Creager, 2016; Graves and Donaghue, 2020). Many studies have linked chronic increases in *O*-GlcNAc associated with diabetes and nutrient excess to the adverse cardiac dysfunction of diabetes (Clark et al., 2003; Fülöp et al., 2007; Fricovsky et al., 2012; Erickson et al., 2013; Umapathi et al., 2021). *O*-GlcNAcylation is involved in several roles that contribute to the development of CVDs, including increased oxidative stress, vascular dysfunction (e.g., impaired vasodilation, increased atherosclerosis, hypertension, and vascular calcification) (Byon and Kim, 2020). This section will focus on the role of *O*-GlcNAcylation in the atherosclerotic cardiovascular disease in diabetes (Tsalamandris et al., 2019). DM2, which is a chronic systemic disease of multifactorial origin characterized by hyperglycemia associated with insulin resistance, has been closely related to a low-grade systemic inflammation (LGSI), which could be present before detectable metabolic alterations; during LGSI, various tissues such as adipose, liver, and muscle suffer from infiltration and activation of immune cells that produce cytokines such as interleukin 6 (IL-6) and tumor necrosis factor (TNF), inducing chronic LGSI with repercussions on all tissues, including the pancreatic tissue and heart tissue (Spranger et al., 2003; Böni-Schnetzler and Meier, 2019). The inflammatory process in β-pancreatic cells can be mediated mainly by both the activation of TLRs mediated by molecular patterns associated with damage and advanced glycation end products (Kong et al., 2017), as well as by the activation of the inflammasomes (Kong et al., 2017). The result is insulitis characterized by the release of pro-inflammatory cytokines and chemokines, with the subsequent activation of macrophages *in situ* and the recruitment of cells of the immune line. Other soluble mediators such as IL-12, IL-17, and NADPH oxidase-1 are also related to islet inflammation (Donath et al., 2013; Eguchi and Manabe, 2013; Imai et al., 2013; Böni-Schnetzler and Meier, 2019).

In the same sense, during LGSI, insulin resistance can be generated by the activation of signaling cascades of Janus kinases (JAK) and activating signals of transcription (STAT), which are related to a variety of cytokines, hormones, and growth factors that regulate homeostatic processes including hematopoiesis, stem cell maintenance, growth, development and differentiation in cells of immune lineage (Dodington et al., 2018); however, the chronic activation of JAK/STAT underlies obesity (Sun and Karin, 2012; Kern et al., 2018). In obesity, there are circulating levels of IL-6 that, when binding to its receptor, induces phosphorylation and activation of the JAK2/STAT3 pathway, which transcriptionally regulates its suppressor, the suppressor of cytokine signaling (SOCS3) (Heinrich et al., 2003), which regulates the signaling generated by JAK/STAT. The high signaling by IL-6 generates high levels of SOCS1 and SOCS3 in tissues sensitive to insulin (Ueki et al., 2004), which impairs the action of this hormone by binding with the IRS-1 and IRS-2, inducing the ubiquitination and degradation of the latter, leading to insulin resistance (Heinrich et al., 2003), a state of hyperglycemia with subsequent compensatory hyperinsulinemia, followed by wasting and claudication and apoptosis of β-pancreatic cells, insulitis, decreased insulin levels, and the generation of DM2. Inflammation is present also in type 1 diabetes mellitus (DM1); it has been demonstrated that both humoral and cellular immunity is involved in the pathogenesis of DM1 (Bottazzo et al., 1974; Willcox et al., 2009; Atkinson et al., 2012). Some theories about the predisposition to DM1 involve environmental factors in early life (e.g., infections, nutrition, and chemicals capable of activating the autoimmune response), although this still needs clarification (Atkinson and Eisenbarth, 2001; Hyttinen et al., 2003). Nevertheless, the mechanism by which diabetes promotes CVD risk is poorly understood. However, there is evidence that monocytes and macrophages play a critical role in all stages of atherosclerosis because the diabetic microenvironment influences the phenotype of these cells (Kanter et al., 2020). In this sense, *O*-GlcNAcylation could be a critical factor in the regulation of inflammation in CVDs. As described above, chronic, low-grade inflammation associated with obesity and diabetes results from the infiltration of adipose and vascular tissue by immune cells and contributes to CVDs (Li et al., 2019); it has been demonstrated that the levels of inflammatory cytokines are increased in patients with HF (Fiordelisi et al., 2019). HF is associated with metabolic changes that cause a progressive impairment of cardiac muscle (Sorriento and Iaccarino, 2019). In this sense, it is known that the eNOS plays a critical role in regulating the cardiovascular system because eNOS is a calcium/calmodulin-dependent enzyme that catalyzes the synthesis of NO, a critical mediator of vascular homeostasis (endogenous vasodilator) (Takemoto et al., 1997; Erkens et al., 2017). The eNOS activity is related to the crosstalk between phosphorylation and *O*-GlcNAcylation (Heiss and Dirsch, 2014). Recent studies show that overexpression of OGT by addition of GlcN (5 mM) and/or LPS (0.1 μg/ml) inhibits NF-κβ reporter activity and inducible nitric oxide synthase (iNOS) promoter activity and suppresses the GlcN-mediated inhibition of NF-κβ/iNOS transcription (Hwang et al., 2013). However, OGT functions as a transcriptional repressor during inflammatory stimulation; this activity functions in concert with other transcriptional complexes to inhibit over-activation or adjust the inflammatory response (Hwang et al., 2013). Likewise, deletion of OGT in mice developed an intensified effect of LPS (100 ng/ml) on NOS2 expression and cytokine production, indicating that *O*-GlcNAcylation may restrain inflammatory processes (Al-Mukh et al., 2020). Increased *O*-GlcNAcylation induced by GlcN (5 mM) and Thiamet G (0.1 µM) (O-GlcNAcase enzyme inhibitor) has a protective effect against TNF-α-induced expression of iNOS protein in aortic segments (Hilgers et al., 2012). TNF-α (10 ng/ml) induces impairment in contractility and endothelium-dependent relaxation in cultured rat aortic rings (Hilgers et al., 2012). *O*-GlcNAcylation of A20 also known as tumor necrosis factor α-induced protein 3, protects against inflammation-induced vascular injury, negatively regulating NF-κβ signaling cascades in TNF-α-treated (10 ng/ml) vascular smooth muscle cells in culture and acutely injured arteries (Yao et al., 2018). Another study in cardiomyocytes demonstrated that after hemorrhagic trauma shock-induced in fasted, male adult rats, the administration *in vivo* of GlcN, as follows, 0.5 ml of 150 mM bolus of GlcN 30 min after the onset of resuscitation followed by 2 ml of 150 mM of GlcN mixed with resuscitation solution, attenuates the activation of NF-κβ in the heart, and was associated with improved cardiac function, decreased systemic inflammatory response, and increased tissue *O*-GlcNAc levels (Zou et al., 2009). Also, GlcN (5 mM) for 30 min followed by LPS (2 µg/ml) induces IκB-α phosphorylation and iNOS expression in macrophages *in vitro*, which has an anti-inflammatory effect in circulating mediators (Zou et al., 2009). Another example of how *O*-GlcNAcylation could regulate secreted extracellular matrix proteins as well as cell-surface proteins is by thrombospondin-1 which is a potent proatherogenic and anti-angiogenic protein, and its expression level is enhanced in the plasma and walls of the large blood vessel in a model of hyperglycemia-induced atherosclerosis (Hoffmann et al., 2012); recently, it was demonstrated that thrombospondin-1 is *O*-GlcNAcylated and regulated by Cr3+ promoting atheroprotective effects (Ganguly et al., 2017).

Another unusual way by which *O*-GlcNAcylation has been shown to be involved in atherosclerotic CVDs in individuals with diabetes is in the design of GPCR peptide-agonists (Zhang and Xie, 2012) because *O-*GlcNAc improves their stability and *in vivo* activity. For example, it is known that glucagon-like peptide 1 receptor agonists prevent the incidence of atherosclerotic CVDs in people with diabetes (Lee and Jun 2016; Helmstädter et al., 2020). Specifically, exendin-4, a glucagon-like peptide 1 receptor agonist, has been shown to reduce atherosclerosis and monocyte adhesion to aortas, as well as the expression of the inflammatory gene in macrophages in mice, likely by increasing cAMP levels in these cells (Arakawa et al., 2010); therefore, glucagon-like peptide 1 receptor activation has anti-inflammatory effects, direct effects on the heart (Drucker, 2016). Levine et al. suggest that “*O*-GlcNAcylation should be part of the therapeutic peptide tool kit” (Levine et al., 2019) because *O*-GlcNAc could bring “remote stabilization” to peptides. It is important to say that, in the human body, the stability of secreted proteins is naturally increased among others by endogenous glycosylation, like *N-* and mucin *O*-linked glycosylation (Madsen et al., 2020). However, these structures are large and complex, making their therapeutic implementation more difficult, so that *O*-GlcNAcylation could represent an alternative in the design of therapeutic peptides in CVDs.

## Discussion and Future Directions

Technological advances have allowed the study of the glycome and understand its complexity and implication in living beings. The structure and size of glycans branching may vary according to different factors such as age, biological process, genetic defects, sex, lifestyle, or diseases (e.g., cardiovascular diseases); for this reason, glycosylation is a process that could provide important information (Raman et al., 2005). Although it seems that *O*- and *N*-glycosylation are different from *O*-GlcNAcylation, all of them may have variations in the same microenvironmental cellular, which make these PTMs share something in common. For example, the *O*- and *N*-glycosylation of plasma membrane proteins controls folding, transduction, stability, and interaction with partners, whereas O-GlcNAcylation should indirectly affect behaviors of *N*- and *O*-glycoproteins, such as CD36, which facilitate the transport of fatty acids (Luiken et al., 2016). Nevertheless, is not fully clear how changes in *N*- and *O*-glycosylation in other receptors like TLR4 or insulin receptor are involved in cardiovascular disease. Biwi et al. present an excellent and exhaustive review about the possibilities on the control of complex glycosylations at the various levels that *O*-GlcNAcylation can exert (Biwi et al., 2018). The cardiovascular system also has intracellular signalling pathways regulated by different post-translational modifications, including *O*-GlcNAcylation, which promotes adequate responses to extracellular stimuli and signalling transduction. In conclusion, to carry out treatment and diagnosis of CVDs, it is necessary to bear in mind the role of glycosylation at different levels, reception of signal, signal transduction, and exogenous molecules or agonist that stimulate the heart and vascular wall cells.

We show in this review how glycosylation in the heart and vascular wall is involved in the response of the cardiovascular system to changes in the microenvironment through different extracellular stimuli, such as molecular patterns associated with damage such as extracellular HSP60 (Kim et al., 2009; Zindel and Kubes, 2020), and the recently described molecular patterns associated with lifestyle, for example ox-LDL (Glatz et al., 2013; Zindel and Kubes, 2020), or possibly the existence of molecular patterns associated with homeostasis that are recognized by different receptors, for example CD36 and TLR4 (Frantz et al., 1999), intervening in the development of sterile inflammation. The expansion of this knowledge will facilitate the design of peptides, proteins, or agonists modulated by glycosylation (Tamburrini et al., 2020) as a therapeutic tool for the control of CVDs. We think that there are a great variety of unknown and potentially glycosylated extracellular stimuli that have simultaneous effects in different conditions, such as in ischemia/reperfusion or as in low-grade inflammation in diabetes, and possibly in aging, during exercise, and different lifestyles. An opportunity for the future will be to identify and evaluate therapeutic lifestyle strategies that might directly influence changes in glycosylation that could be beneficial to cardiovascular health.

## Abbreviations

Apo, Apolipoprotein; D-GlcN, D-glucosamine; Fru, Fructose; GalN, Galactosamine; GalNAc, *N*-Acetylgalactosamine; GFAT, Glutamine fructose-6-amidotransferase; Glc, Glucose; GlcN, Glucosamine; GlucNAc, *N*-Acetylglucosamine; GLUT, Glucose transporter; GPCRs, G protein-coupled receptors; GPs, Glycan peaks; HBP, Hexosamine biosynthetic pathway; HDL, High density lipoprotein; HF, Heart failure; iNOS, Inducible nitric oxide synthase; IRS1, Insulin receptor substrate 1; LDL, Low density lipoprotein; LPS, Lipopolysaccharide; MAPK, Mitogen-activated protein kinase; NF-κβ, Nuclear factor enhancer of the kappa light chains of activated B cells; OGA, *N*-Acetyl beta-D-glucosaminidase (NAGase); OGT, *O*-Linked N-Acetylglucosamine (GlcNAc) Transferase; SREBP1, Sterol-regulatory element-binding protein 1; TC, Total cholesterol; TG, Triglycerides; TNF-α, Tumor necrosis factor-α; UDP-GlcNAc, Uridine diphosphate-*N*-acetylglucosamine; β1AR, β1-adrenoceptor.
